# Time-programmable pH: decarboxylation of nitroacetic acid allows the time-controlled rising of pH to a definite value†

**DOI:** 10.1039/d1sc01196k

**Published:** 2021-04-21

**Authors:** Daniele Del Giudice, Emanuele Spatola, Matteo Valentini, Cecilia Bombelli, Gianfranco Ercolani, Stefano Di Stefano

**Affiliations:** Dipartimento di Chimica, Università degli Studi di Roma “La Sapienza” P.le A. Moro 5 I-00185 Rome Italy stefano.distefano@uniroma1.it; ISB-CNR Sede Secondaria di Roma – Meccanismi di Reazione c/o Dipartimento di Chimica, Università degli Studi di Roma “La Sapienza” P.le A. Moro 5 I-00185 Rome Italy; Dipartimento di Scienze e Tecnologie Chimiche, Università di Roma Tor Vergata Via della Ricerca Scientifica 00133 Rome Italy ercolani@uniroma2.it

## Abstract

In this report it is shown that nitroacetic acid **1** (O_2_NCH_2_CO_2_H) can be conveniently used to control the pH of a water solution over time. Time-programmable sequences of the kind pH_1(high)_–pH_2(low)_–pH_3(high)_ can be achieved, where both the extent of the initial pH jump (pH_1(high)_–pH_2(low)_) and the time required for the subsequent pH rising (pH_2(low)_–pH_3(high)_) can be predictably controlled by a judicious choice of the absolute and relative concentrations of the reagents (acid **1** and NaOH). Successive pH_1(high)_–pH_2(low)_–pH_3(high)_ sequences can be obtained by subsequent additions of acid **1**. As a proof of concept, the method is applied to control over time the pH-dependent host–guest interaction between alpha-cyclodextrin and *p*-aminobenzoic acid.

## Abstract 2

Predictable and time-programmable sequences of the kind pH_1(high)_–pH_2(low)_–pH_3(high)_ in water solution are obtained by a judicious choice of the concentration of nitroacetic acid undergoing decarboxylation.

## Graphical abstract

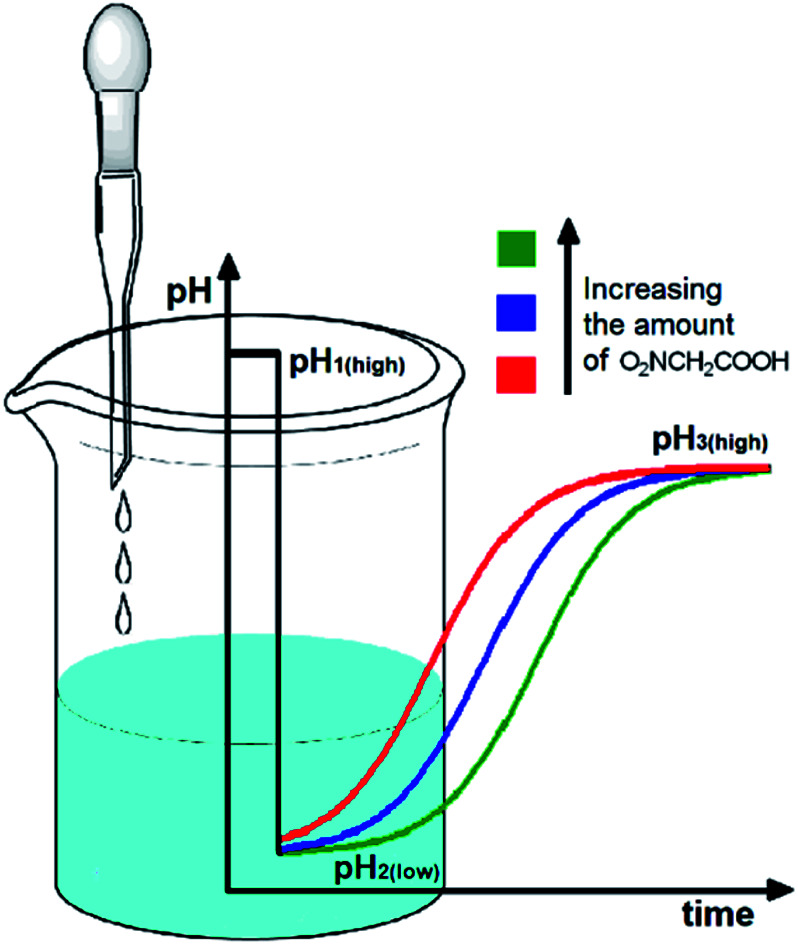

## Introduction

Bio-systems are characterized by a high level of complexity, which allows fine control of the associated functions.^1^ Among different kinds of regulation, control over time is essential to ensure the optimal operation of bio-chemical networks in living systems. Regulation of gene expression and protein synthesis are well-known examples where time-controlled changes of the concentration of some chemical species modulate the outcomes of specific processes occurring in the cell.^2^ Of course, such kind of control is also desirable in the case of artificial chemical systems, which are typically much less sophisticated and feature a lower number of modulable properties.

Since biochemistry has evolved in water, one of the ancestral tools used by nature to control the chemical properties of a compound, or a group of compounds, is pH. For this very reason, the operation of many artificial systems and materials inspired by nature, and aspiring to have life-like properties, is based on pH changes affecting protonation or deprotonation of the involved molecular structures.^3^ DNA-based molecular machines whose motions are guided by pH changes are appropriate examples.^4^ In this case, a time-predictable change of pH would guarantee a full control on the molecular motions of the machine over time. The same holds for chemical architectures or aggregates, which assemble and disassemble under the influence of pH variations.^5^ In one case, the time controlled assembly/disassembly of a micellar system was elegantly achieved through programmed variations of pH (up–down sequences) obtained by a judicious choice of the hydrolysis conditions of a series of ester compounds of abiotic nature.^6^ In other cases, the time controlled assembly/disassembly was made possible by the presence of enzymes, which allow the required pH variations.^7^ Alternatively, pH variations effecting aggregation/disaggregation phenomena have also been achieved by bubbling gaseous CO_2_ in solution.^8^ More in general, a number of systems including dissipative systems^9,10^ could take advantage from time-programmable control of pH.

Lately, some activated carboxylic acids such as 2-cyano-2-phenylpropanoic acid,^10,11^ and its derivatives,^12^ or trichloroacetic acid,^13^ have been successfully used to promote time controlled cycles of motions of molecular machines, both switches and motors, in organic media. In all cases, the movements within the molecular machine were due to sequential protonation and deprotonation of a nitrogen base present in the machine skeleton. Initially, the carboxylic acid donates the proton to the nitrogen base of the molecular machine that passes from state A to state B with a consequent large amplitude motion. The subsequent decarboxylation of the resulting carboxylate generates a strong base, which is able to take back the proton from the protonated molecular machine. As a consequence, the state A of the latter is restored with a second large amplitude movement that closes the cycle of motion.^9^

Taking inspiration from these studies, which were all carried out in organic solvents, we wanted to devise a chemical system able to modulate the pH in water over time. After some preliminary search,^14^ we resorted to nitroacetic acid^15^ (O_2_NCH_2_CO_2_H) as putative carboxylic acid to achieve such pH control. Hereafter we report the results of our investigation.

## Results and discussion

The idea at the basis of the present investigation is to attain a time-controlled pH variation of a water solution by means of a buffer species, which changes nature over time. Examination of the literature suggested that nitroacetic acid, **1**, could be used to this end. It is a diprotic acid (p*K*_a1_ = 1.48, and p*K*_a2_ = 8.90, in water at 23.5 °C and *μ* = 0.10 M),^15^ whose singly deprotonated form, **2**, in contrast to the parent acid **1** and the doubly deprotonated form, **3**, smoothly decarboxylates in water at room temperature (first-order rate constant *k* = 0.121 min^−1^ at 23.5 °C and *μ* = 0.10 M).^15^

**Scheme 1:**
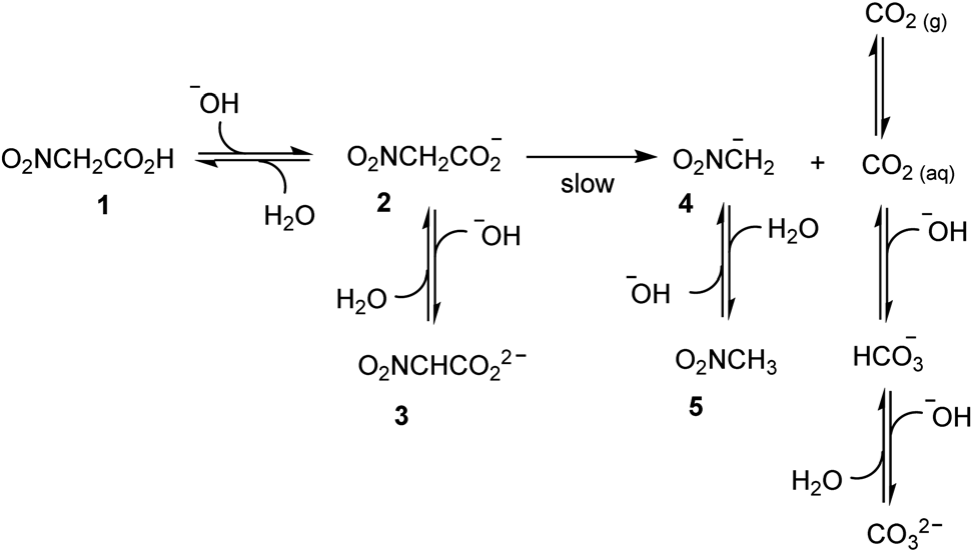
Expected scheme for decarboxylation of nitroacetic acid **1**.

In an ideal, prototypal experiment, a 0.010 M water solution of NaOH is prepared. To this, solution one or more mol equiv. of nitroacetic acid are added so that the pH rapidly changes from basic to acidic. At this point, the mono-anion **2** begins to irreversibly decarboxylate, and the resulting nitromethide ion, **4**, (p*K*_a_ = 10.28 at 20 °C and *μ* = 0.50 M)^16^ takes back the proton from the solvent to eventually raise the pH of the solution back to basic values. As illustrated in Scheme 1, however, the pH is affected not only by the aforementioned processes, but also by the formation of carbon dioxide. If the experiment is carried out by bubbling Ar (as well as N_2_ or air) into the solution by means of a capillary connected to a gas cylinder, one can avoid carbon dioxide oversaturation of the solution. Under this condition, the concentration of carbon dioxide remains at the constant value of its solubility in water, *i.e.* 1.63 × 10^−5^ M at 20 °C, during the entire course of the experiment.§§The concentration of CO_2_ in the atmosphere is 415 ppm (value taken from the website https://www.co2.earth on January 18^th^, 2021), meaning that its partial pressure is 4.15 × 10^−4^ atm. Its solubility in water at 20 °C is 0.0393 mol per kg water, at a partial pressure of 1 atm CO_2_ (see ref. 17). At such low pressure Henry's law is followed (see ref. 17), and thus the molar concentration of CO_2_ in water at 20 °C can be calculated as the product of its solubility times its partial pressure in the atmosphere.^17^ It is known, that more than 99% of CO_2_ exists in solution as the dissolved gas and less than 1% as carbonic acid H_2_CO_3_, which partially dissociates to give HCO_3_^−^ and CO_3_^2−^ ions. Owing to the difficulty of differentiating between CO_2_ and H_2_CO_3_ in solution, the apparent first ionization constant, defined as [HCO_3_^−^][H_3_O^+^]/([CO_2_] + [H_2_CO_3_]), is generally considered. Accordingly, carbon dioxide can be treated as a diprotic acid with an apparent p*K*_a1_ = 6.35, and a p*K*_a2_ = 10.33 at 25 °C.^17^

**Fig. 1:**
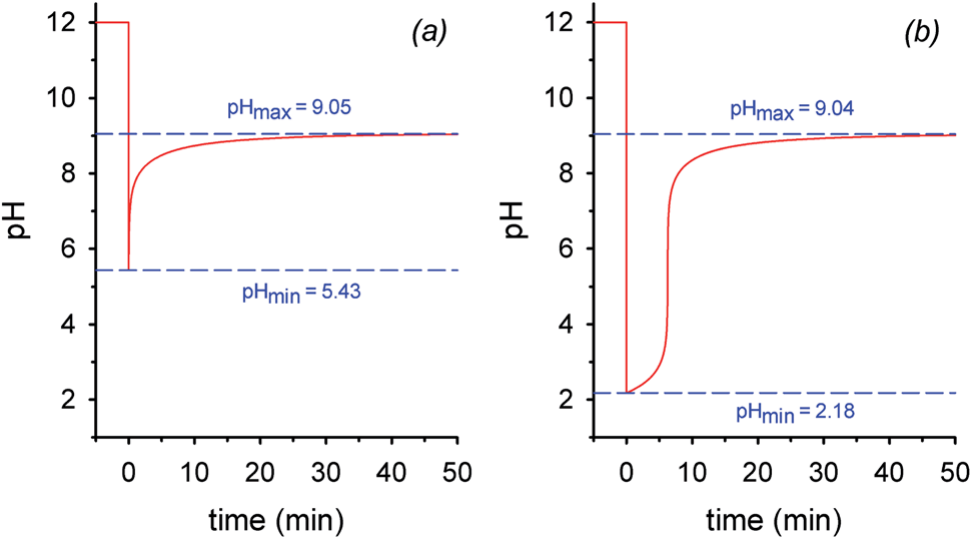
Simulated plots of pH *vs.* time: (a) 1 mol equiv. of nitroacetic acid **1** is added to a solution of 0.010 M NaOH at time *t* = 0; (b) 2 mol equiv. of nitroacetic acid **1** are added to a solution of 0.010 M NaOH at time *t* = 0.

By assuming that all the equilibria in Scheme 1 are much faster than the process of decarboxylation, all the kinetic and equilibrium constants necessary to investigate the dynamics of the system are known. However, owing to its formidable complexity, the system is not amenable to a simple analytic solution. For this very reason, in order to forecast the behavior of the system, we recurred to numerical integration using the program COPASI (COmplex PAthway SImulator), which is an open-source software application for simulation and analysis of chemical networks and their dynamics.^18^ We simulated two experiments: in the first experiment (a), 1 equiv. of nitroacetic acid **1** is added to an initial 0.010 M NaOH solution (pH = 12), while, in the second experiment (b), 2 equiv. of nitroacetic acid **1** are added to the same initial NaOH. The kinetics of pH changes for the two experiments are reported in Fig. 1, whereas the kinetics of the concentration changes of all the species appearing in Scheme 1 are reported in the ESI (pages S3–S7).†

The kinetics in Fig. 1 are readily understood. In experiment (a), nitroacetic acid, **1**, is completely neutralized by NaOH, and the resulting nitroacetate, **2**, solution shows an initial pH of 5.43. However, this solution is not buffered, and thus the progress of the decarboxylation reaction causes a steep pH jump. After the initial jump, the pH changes more slowly because of the buffer pairs present in solution (**2**/**3**, **5**/**4**, CO_2 aq_/HCO_3_^−^, and HCO_3_^−^/CO_3_^2−^) up to the final pH value of 9.05 which is determined by the latter three buffer pairs. The final dominant species in solution are nitromethane, **5**, and the hydrogen carbonate anion. In experiment (b), nitroacetic acid, **1**, is partially neutralized by NaOH, however since **1** is a relatively strong acid, initially the ratio [**2**]/[**1**] is larger than 1 (actually the ratio is about 5) and the resulting pH = 2.18. Until the undissociated acid **1** is present, the solution is buffered and the progress of the decarboxylation reaction causes a limited pH increase. When the acid **1** is completely neutralized, the solution is no more buffered and a steep pH jump ensues. After the jump, the pH increases more slowly as in experiment (a) and for the same very reasons. It is noteworthy that the final pH value is very similar in the two experiments. Comparing the two kinetic profiles, it is immediately apparent that the experiment with excess acid benefits from a larger pH jump, and therefore the presence of excess acid is more suitable for making molecular machines or other dissipative systems work. Moreover, Fig. 1 suggests that by increasing the excess acid, the time necessary to reach the pH jump can be modulated at will. Thus, a system based on nitroacetic acid could be exploited for chemically programming subsequent pH changes of the kind pH_1(high)_–pH_2(low)_–pH_3(high)_. The choice of the absolute and relative initial concentrations of NaOH and nitroacetic acid dictates values of pH_1_ and pH_2_.

**Fig. 2:**
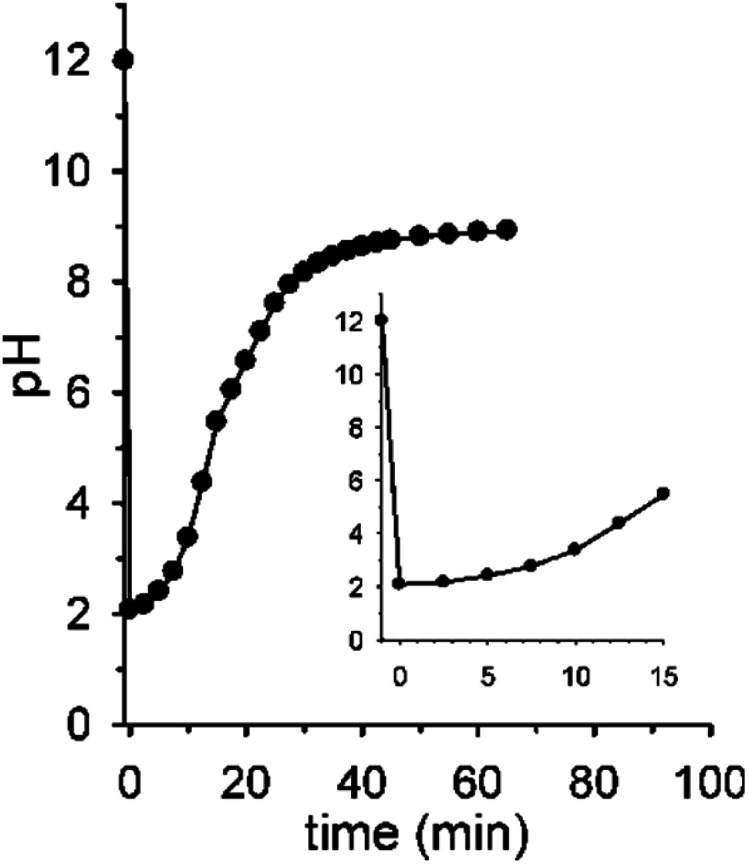
Reaction between 0.010 M NaOH and 0.020 M nitroacetic acid **1** (*T* = 20 °C, *μ* = 0.50 M). The point at pH = 2.05 has been recorded immediately after the addition of **1**. The curve is a guide to the eye.

Fig. 2 shows the real version of one of the prototypal experiments previously designed. Here, the pH of an initial 0.010 M NaOH solution is followed over time (*T* = 20 °C, *μ* = 0.50 M with NaCl¶¶Reaction time and profile do not change if ionic strength is decreased by a factor of 5 (NaCl 0.100 M).) by means of a glass microelectrode. At *t* = 0 pH is 12. When 2 mol equiv. of **1** are added, the pH immediately drops to about 2.05 and, subsequently, starts to rise up again drawing a sigmoidal curve. After 30 min the curve tends to sit down and at *t* = 60 min a pH of 9.0 is read (the kinetic profile described by the experimental points is highly reproducible). The initial and the final pH values are in good agreement with those predicted by the simulation experiment, however the experimental profile is less steep than the theoretical one (compare profiles in Fig. 2 and 1b). This behavior very likely depends on the difficulty for the glass microelectrode to follow the pH changes in solution in real time, probably because of the formation of gas microbubbles over its surface.||||A delayed answer of the glass electrode was effectively proved by following the pH evolution of a solution in which 0.010 M NaOH and 0.020 M acid **1** were reacted in the presence of bromocresol green. The end point was visually detected by the color change of the indicator a non-negligible time before it was recorded by the electrode pH reading.

Interestingly, the initial and final pH values of two runs carried out under the same conditions, but bubbling either argon or air in solution, were the same (see Fig. S8†), supporting the assumption that in both cases the solution is saturated by CO_2_ throughout the experiment.

### Time-controlled rising of the pH to a definite value

**Fig. 3:**
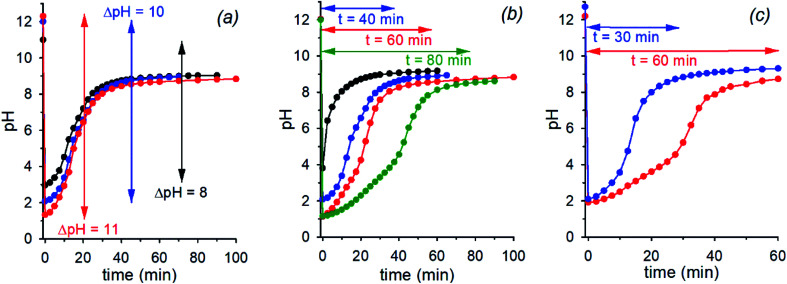
(a) pH time evolution of 0.020 M (red trace), 0.010 M (blue trace) and 0.0010 M (black trace) NaOH solution after addition of 0.040 M, 0.020 M and 0.0020 M acid **1** (H_2_O, *T* = 20 °C, *μ* = 0.50 M), respectively. (b) pH time evolution of a 0.010 M NaOH solution after addition of 0.010 M (black trace), 0.020 M (blue trace), 0.030 M (red trace) and 0.040 M (green trace) acid **1** (H_2_O, *T* = 20 °C, *μ* = 0.50 M). (c) pH time evolution of 0.010 M and 0.036 M NaOH solution after respective addition of 0.026 M (red trace) and 0.060 M (blue trace) acid **1** (H_2_O, *T* = 20 °C, *μ* = 0.50 M). Curves are a guide to the eye.

The initial pH jump of a programmed sequence of the kind pH_1(high)_–pH_2(low)_–pH_3(high)_ can be modulated by a careful choice of the absolute and relative concentrations of the base (NaOH) and the acid (nitroacetic acid **1**). In a series of experiments in which the molar ratio between NaOH and acid **1** was fixed to 1 : 2, change of the absolute concentrations of NaOH and acid **1** allowed different initial pH jumps as shown in Fig. 3a. In the case of [NaOH]_o_ = 0.020 M and [**1**]_o_ = 0.040 M, evolution of pH is 12.3–1.3–8.9 in 40 min (initial ΔpH = 11, red trace), in that of [NaOH]_o_ = 0.010 M and [**1**]_o_ = 0.020 M, it is 12.0–2.0–9.0 in 40 min (initial ΔpH = 10, blue trace), and eventually in that of [NaOH]_o_ = 0.0010 M and [**1**]_o_ = 0.0020 M, it is 11.0–3.0–9.0 in 40 min (initial ΔpH = 8, black trace). Thus, different initial pH jumps are observed followed by very similar time evolutions to a definite value of about 9.

On the other hand, different time-programmable evolutions after the initial pH jump were achieved by fixing the initial concentration of the base and varying the amount of added acid **1**. In Fig. 3b the case in which the [NaOH]_o_ is fixed to 0.010 M and the amount of added acid **1** is varied (1 mol equiv. black trace, 2 mol equiv. blue trace, 3 mol equiv. red trace and 4 mol equiv. green trace) is reported. It is clear that the time required to reach the plateau value, round about 9 in the different cases, increases on increasing the initial concentration of acid **1**, which can be varied at will. It is noteworthy that once the excess acid is over, all the kinetic profiles are practically superimposable. Interestingly, although the time needed to reach the plateau value is more or less the same, when 1 or 2 mol equiv. of acid **1** are added to 0.010 M NaOH (black and blue traces of Fig. 3b, respectively), the pH time-profiles of the two solutions predictably and strongly diverge for the reasons discussed in the previous section.**In an additional series of runs, 0.015 M **1** was added to NaOH of varying concentration, namely, 0.0075, 0.015, 0.030 and 0.050 M. The results obtained, which are in line with expectation, are reported at page 8 of the ESI (Fig. S7).†

Eventually, there is a chance to adjust the concentrations of NaOH and acid **1** in order to obtain exactly the same pH (pH_2(low)_) after addition of **1**, and regulate the time necessary to reach the final pH. This kind of control is illustrated in Fig. 3c that reports two experiments with specific concentrations of NaOH and **1** (0.010 M NaOH + 0.026 M **1** and 0.036 M NaOH + 0.060 M **1**, red and blue trace, respectively) aimed at obtaining the same pH_2(low)_ = 2.

**Fig. 4:**
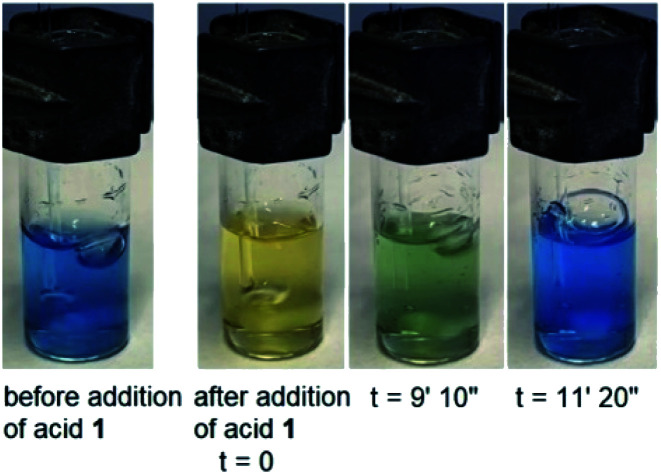
Selected pictures of a 0.010 M NaOH solution before and after addition of 0.020 M acid **1** in the presence of bromocresol green (p*K*_a_ = 4.8). Second pictures from the left (*t* = 0) were taken immediately after addition of acid **1** (see ESI page S10 for related movie†).

The effectiveness of nitroacetic acid **1** in promoting a time-programmed pH_1(high)_–pH_2(low)_–pH_3_ = 9 sequence of a water solution can also be visually illustrated using classical pH indicators. Fig. 4 shows selected pictures taken at different times of a 0.010 NaOH solution before and after addition of 0.020 M acid **1** in the presence of bromocresol green (p*K*_a_ = 4.8). The corresponding movie can be found in the ESI page S10,† together with the movie related to the same experiment carried out in the presence of methyl red (p*K*_a_ = 5.1), see ESI page S11.†

**Fig. 5:**
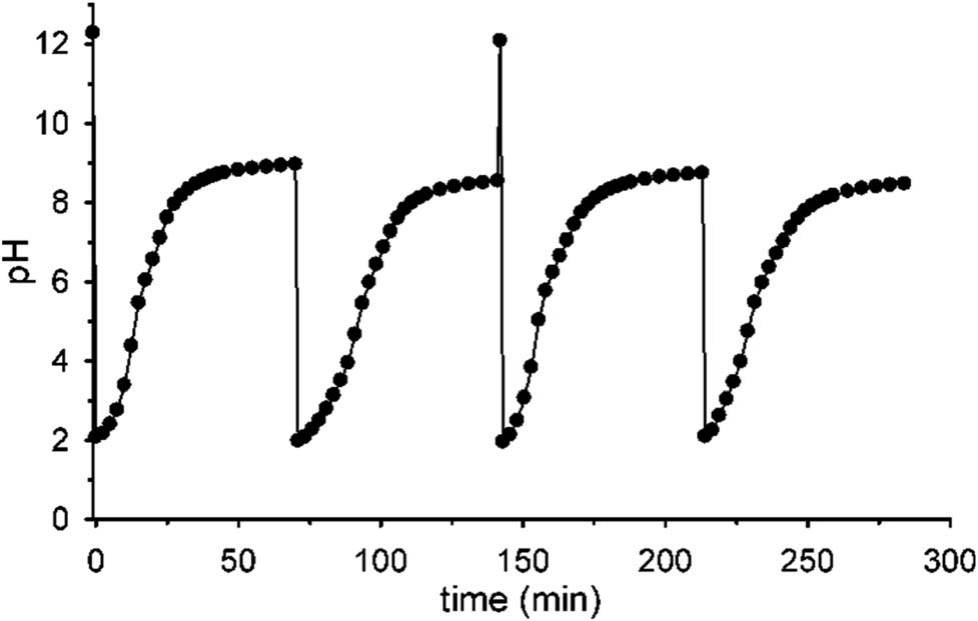
pH cycles achieved by successive additions of nitroacetic acid. To the initial solution (0.010 M NaOH), aliquots of acid **1** are subsequently added to set the pH at 2 and trigger two pH cycles (12–2–9). After these two cycles, the pH was again adjusted to 12 by adding the required amount of NaOH and two more cycles were triggered with subsequent addition of acid **1** (H_2_O, *T* = 20 °C, *μ* = 0.50 M). Curves are a guide to the eye.

Fig. 5 shows that it is possible to repeat more times the sequences pH_1(high)_–pH_2(low)_–pH_3_ = 9 by successive additions of nitroacetic acid. After every two shots, it is convenient to reset the initial pH (pH_1_) by adding the required amount of NaOH in order to have a full efficiency of the system.††††If NaOH is not added after the second pulse of acid **1**, the system loses efficiency and doesn't reach the final pH = 9 anymore, probably due to the accumulation of reaction products (see Scheme 1) that someway affect the decarboxylation process (see Fig. S10 in the ESI†).

### A proof of concept application

**Scheme 2:**
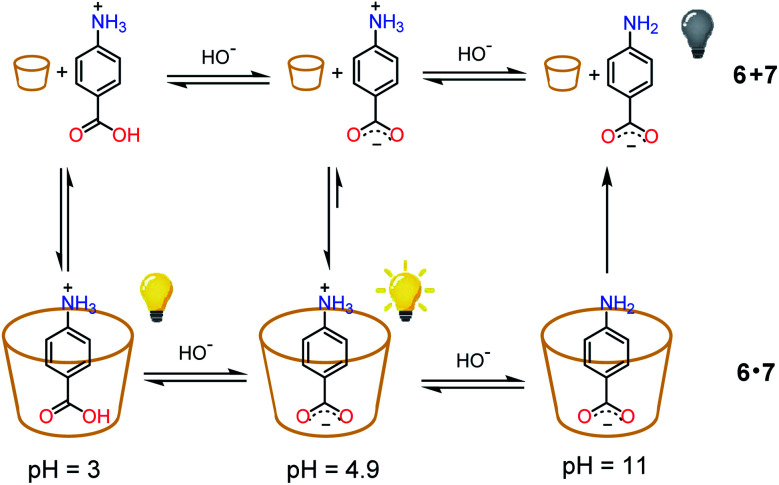
State of charge of **7** at different pHs and corresponding affinity for alpha-cyclodextrin **6**. Relative emission intensities are also schematically shown.

As a proof of concept, the nitroacetic acid driven pH_1(high)_–pH_2(low)_–pH_3(high)_ time-programmed sequence has been applied to the well-known host–guest pH dependent interaction between alpha-cyclodextrin **6** and *p*-aminobenzoic acid **7**, which can be conveniently monitored using a spectrofluorometer.^19^ The charge state of **7** can be easily controlled by adjusting the solution pH. Amino acid **7** is partially positively charged at pH 3.0, neutral (zwitterionic) at intermediate pH and negatively charged at pH 11.0.‡‡‡‡p*K*_a1_ and p*K*_a2_ of *p*-aminobenzoic acid are 2.39 and 4.91, respectively (M. S. K. Niazi and J. Mollin, *Bull. Chem. Soc. Jpn.*, 1987, **60**, 2605–2610), and are expected to considerably change in the presence of excess cyclodextrin. For example, complexation into beta-cyclodextrin, which is a weaker binder than alpha-cyclodextrin, sensibly enhances the p*K*_a2_ (Δp*K*_a_ = 1.5) of *p*-aminobenzoic acid. In the presence of excess **6**, the zwitterionic form of **7** is strongly bound by the cavity of cyclodextrin and consequently the fluorescence emission is high (see Scheme 2).^19^ The binding becomes weaker when **7** is positively charged (lower pH). Under these conditions it is partially released with a consequent, moderate quenching of the fluorescence emission (Scheme 2). Binding is even weaker for the negatively charged form of **7** (higher pH), and the emission becomes definitely less efficient (Scheme 2).^19^ Thus, fluorescence variations are most largely due to the extent of the inclusion of **7** into **6**.

**Fig. 6:**
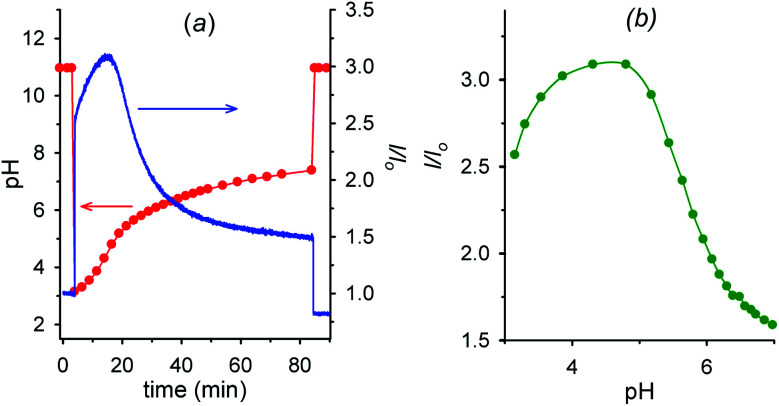
(a) Time dependence of pH and fluorescence emission intensity (*λ*_exc_ = 282 nm, *λ*_em_ = 338 nm) of 0.0050 mM aminobenzoic acid **7** in the presence of 2.0 mM alpha-cyclodextrin **6** (1.0 mM NaOH, *μ* = 0.50 M; *T* = 20 °C) before and after the addition (at *t* = 4 min) of 2.0 mM nitroacetic acid **1**. At *t* = 84 min the amount of NaOH required to restore pH 11 was added. (b) Corresponding *I*/*I*_o_*vs.* pH from *t* = 4 min to *t* = 84 min obtained from graphs in Fig. 6a. Red and green curves are a guide to the eye.

Fig. 6a shows the fluorescence and the corresponding pH variations as a function of time when acid **1** (2.00 mM) is added to a solution containing NaOH (1.00 mM), **6** (2.00 mM) and **7** (0.0050 mM). Before addition of acid **1**, the solution pH is 11.0 (*t* = 0, red trace), **7** is in its negative form and is largely outside the cavity of cyclodextrin, and consequently the fluorescence emission is low (*t* = 0, blue trace). Immediately after the addition of acid **1** (*t* = 4 min), the pH drops to 3.0 and **7**, which is now mostly in its positive form, is more efficiently bound by **6**, resulting in an immediate strong enhancement of the fluorescence.

Then, decarboxylation takes place and pH begins to increase. After 16.5 min from start, at pH 4.9, the concentration of the zwitterion is at its maximum value, concentration of the inclusion complex **6**·**7** is at its maximum too, and, correspondingly, the fluorescence reaches its highest value (see also Fig. 6b). From 16.5 min (pH 4.9) onwards, the pH still rises, and the zwitterionic form of **7** is partially transformed into the negatively charged one. The above complex partially dissociates and the fluorescence decreases again. Interestingly, the fluorescence variation shows an opposite behavior in the absence of cyclodextrin **6** (see Fig. S12†). For a technical difficulty due to the instrumental set up, the experiment was carried out without argon bubbling. This is the reason why the final pH does not rise to the usual value of 9 but reaches 7.4 at 84 min from start. Furthermore, the p*K*_a_ of the second dissociation of *p*-aminobenzoic acid **7** is strongly enhanced by inclusion into cyclodextrin,^20^ and thus at the end of the monitoring (pH = 7.4), **7** is still partially in its zwitterionic form.§§§§At *t* = 84 min, addition of the required amount of NaOH to reset the pH solution to 11 restored approximately the initial value of the fluorescence. However it is evident that an association–disassociation cycle of the kind **6** + **7** → **6**·**7** → **6** + **7** has been achieved *via* a nitroacetic acid driven time-programmed pH_1(high)_–pH_2(low)_–pH_3(high)_ sequence (namely, 11–3–7.4).

## Conclusions

To sum up we have shown that the decarboxylation reaction of nitroacetic acid **1** can be conveniently used to control the pH of a water solution over time. Predictable and programmable sequences of the kind pH_1(high)_–pH_2(low)_–pH_3_ = 9 can be achieved by a judicious choice of reagent (NaOH and acid **1**) concentrations. In particular, the initial pH jump, pH_1(high)_–pH_2(low)_, and the time required for the subsequent one pH_2(low)_–pH_3_ = 9, can be regulated at will. A simple application of the methodology has been reported as a proof of concept, which hopefully serves as a prelude to general applications in pH-dependent dissipative systems that operate in water.

## Experimental

Nitroacetic acid **1** was prepared as previously described,^21^ and alpha-cyclodextrin **6** and *p*-aminobenzoic acid **7** were purchased from Merck and used as received. pH measurements were carried out with a micro glass electrode 52 08HACH (Ag/AgCl) connected to a Crison pH 25+ pH-meter and the fluorescence measurements were carried out with a Horiba Jobin-Yvon FLUOROMAX 4 spectrofluorometer. For details on experimental executions see ESI.†

## Author contributions

SDS conceived the general design. GE carried out the simulations. SDS, GE, DDG, ES and CB designed the experiments. DDG, ES, MV, and CB performed the experiments. SDS and GE wrote the article.

## Conflicts of interest

There are no conflicts to declare.

## Supplementary Material

SC-012-D1SC01196K-s001
