# Supplementary material for: Time-programmable pH: decarboxylation of nitroacetic acid allows the time-controlled rising of pH to a definite value
Source: Chem Sci. 2021 Apr 21;12(21):7460–6. doi: 10.1039/d1sc01196k (PMC8171335; doi:10.1039/d1sc01196k)
Supplement: SC-012-D1SC01196K-s001 [file SC-012-D1SC01196K-s001.pdf]

Electronic Supplementary Material (ESI) for Chemical Science.
This journal is © The Royal Society of Chemistry 2021

Electronic Supplementary Information for

Time-programmable pH: Decarboxylation of Nitroacetic Acid Allows the Time-controlled Rising of pH to a Definite Value

Daniele del Giudice, Emanuele Spatola, Matteo Valentini, Cecilia Bombelli, Gianfranco Ercolani and Stefano Di Stefano

Content

Experimental Methods S1

Simulated kinetics of 0.010 M NaOH solution added with 1 equiv. of nitroacetic acid **1** S3

Simulated kinetics of 0.010 M NaOH solution added with 2 equivs. of nitroacetic acid **1** S5

Kinetic runs obtained adding 0.015 M acid **1** to 0.0075, 0.015, 0.030 and 0.050 M NaOH S8

pH Monitoring of the Reaction between 0.015 M NaOH and 0.030 M **1** with argon or air bubbling S9

pH Monitoring of the Reaction between 0.010 M NaOH and 0.020M **1** without argon bubbling S9

Decarboxylation followed with bromocresol green S10

Decarboxylation followed with methyl red S11

Three $pH_{1(high)}$-$pH_{2(low)}$-$pH_{3(high)}$ sequence triggered by three subsequent fuel pulses without restoring the initial pH (12) after the first two pulses. S12

A control experiment S13

Comparison between the fluorescence emission vs time profiles of 0.0050 mM **7** during the decarboxylation of 2.0 mM **1** triggered by 1.0 mM NaOH in the presence or absence of **6** S14

Experimental Methods

All the commercially available reactants were used without further purifications. Nitroacetic acid **1** was obtained following a literature procedure.[1]

NaOH and NaCl solutions were prepared using Milli-Q water produced by a Direct-Q3 Millipore apparatus (MERCK KGaA, Burlington, MA, USA) and bubbling argon in the vessel for 15 minutes before use. Nitroacetic acid **1** stock solutions were prepared just before use dissolving 2-20 milligrams of **1** in the proper volume of doubled distilled water, and handled quickly to prevent undesired decarboxylation. All the experiments were carried out at 20°C in a 4 mL (or 8 mL) vial equipped with a capillary connected to a gas cylinder to ensure argon bubbling into the solutions during the measurements. pH was monitored over time with a glass microelectrode 52 08HACH (Ag/AgCl) connected to Crison pH 25+ pHmeter. The final NaCl concentration was always 0.500 M.

Decarboxylation experiments

Decarboxylation kinetics were carried out on a total volume of 2.0 mL. Firstly, the proper volume of 1.00 M NaOH was added to a NaCl solution. Then, the first point was recorded. Eventually, the proper volume of a freshly prepared, concentrated stock solution of nitroacetic acid **1** was added to obtained 2.0 mL of solution with the required concentrations of the components (t = 0). pH variation as a function of time was recorded by reading the pH value on the pH screen at the corresponding time. All the measurements were highly reproducible (repeated three or four times).

Fluorescence measurements

Fluorescence emission kinetics were recorded at room temperature (T = 20°C) on a Horiba Jobin-Yvon FLUOROMAX 4spectrofluorometer (Kyoto, Japan). All collected data were corrected by means of a built-in program in order to counterbalance the decay in sensitivity in the near infrared region and divided by the corrected reference detector. Fluorescence experiments were carried out on solutions with optical density lower than 0.10 to minimize the inner filter effect; samples were prepared immediately before the fluorescence measurements in a quartz cuvette with a 1 cm path-length.

*Decarboxylation kinetics in presence of **6** and **7***

Decarboxylation kinetics in the presence of *p*-aminobenzoic acid **7** and *alpha*-cyclodextrin **6**, were followed simultaneously by means of the pHmeter and the spectrofluorometer. In a 8.0 mL vial, equipped with the microelectrode connected to the pHmeter, the proper amounts of NaOH (stock solution 1.00 M), **7** and **6** were added to a NaCl solution, to give a volume of 5.8 mL. Then, 2.9 mL of the 5.8 mL were transferred into a quartz cuvette lodged inside the spectrofluorometer holder and the pH and fluorescence measurements were started, the former in the vial and the latter in the cuvette. At t = 4 min, equal volumes of a freshly prepared stock solution of nitroacetic acid **1** were simultaneously added to both vial and cuvette (final volumes were 3.0 mL in both cases and in both solutions initial (t = 4 min) concentrations were: $[\mathbf{1}]_o$ = 2.0 mM, $[NaOH]_o$ = 1.0 mM, $[\mathbf{6}]_o$ = 2.0 mM, and $[\mathbf{7}]_o$ = 0.0050 mM, $[NaCl]_o$ = 500.0 mM).

The reaction was followed for 84 min (see Fig. 6a in the main text). The variation of the fluorescence emission (λ_{em} = 338 nm) over time is reported as I/I_0, where I_0 is the fluorescence emission at pH 11 before the addition of **1**, and I is the fluorescence emission at time t.

The above kinetic run was also carried out in the absence of **6**. The related pH and fluorescence vs time profiles (red and orange, respectively) are reported in Fig. S11.

Fig. S12 reports the comparison between the fluorescence profile of Fig. 6a (blue trace, in the presence of **6**) and the fluorescence profile of Fig. S11 (orange, in the absence of **6**).

Simulated kinetics of 0.010 M NaOH solution added with 1 equiv. of nitroacetic acid 1

Kinetics were simulated with the program COPASI[18] by assuming that all the equilibria in Scheme 1 are much faster than the process of decarboxylation, and that the concentration of CO_2 in solution remains fixed to its saturation value throughout the simulated experiment. In Table 1 are summarized the kinetic and equilibrium constants used for the simulation.

Table S1. Summary table reporting the parameters used for drawing the theoretical curves reported in Fig. 1a and 1b and in Figs. S1-S6 (theoretical experiments: [NaOH] =0.010 M + [**1**] = 0.010 M and [NaOH] =0.010 M + [**1**] = 0.020 M).

k (min^{-1})	pK_{a1}	pK_{a2}	pK_a	p$K_{a1}^{CO_2}$	p$K_{a2}^{CO_2}$	$[CO_2]aq^{sat}$ (M)
0.121	1.48	8.90	10.28	6.35	10.33	1.63×10^{-5}

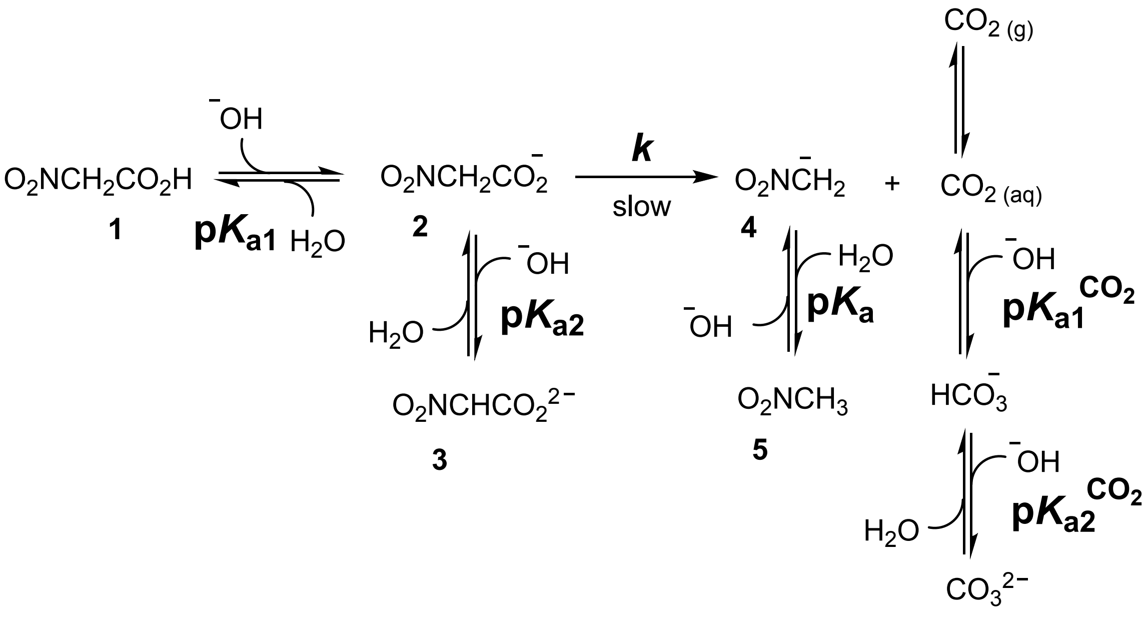

In Fig. 1a is reported the plot of pH vs. time when 1 equiv. of nitroacetic acid **1** is added to a solution of 0.01 M NaOH at time $t = 0$. The kinetics of nitroacetic acid **1**, the monoanion **2**, and the dianion **3** for the title experiment are reported in Fig. S1.

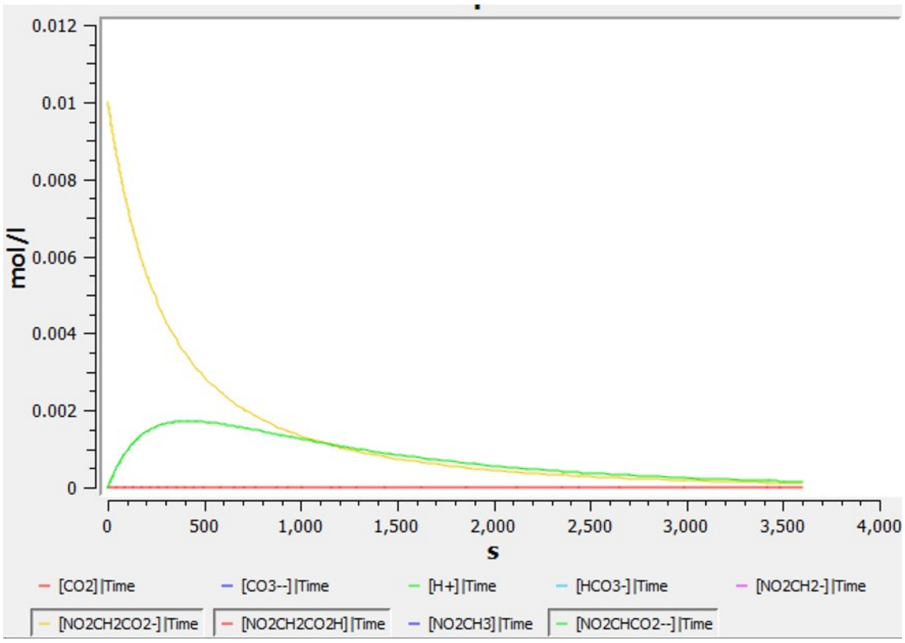

Fig. S1. Plots of the concentrations of nitroacetic acid **1** (red line), the monoanion **2** (yellow line), and the dianion **3** (green line) vs. time when 1 equiv. of nitroacetic acid **1** is added to a solution of 0.01 M NaOH at time $t = 0$.

The kinetics of nitromethane **5**, and its conjugate base **4** for the title experiment are reported in Fig. S2.

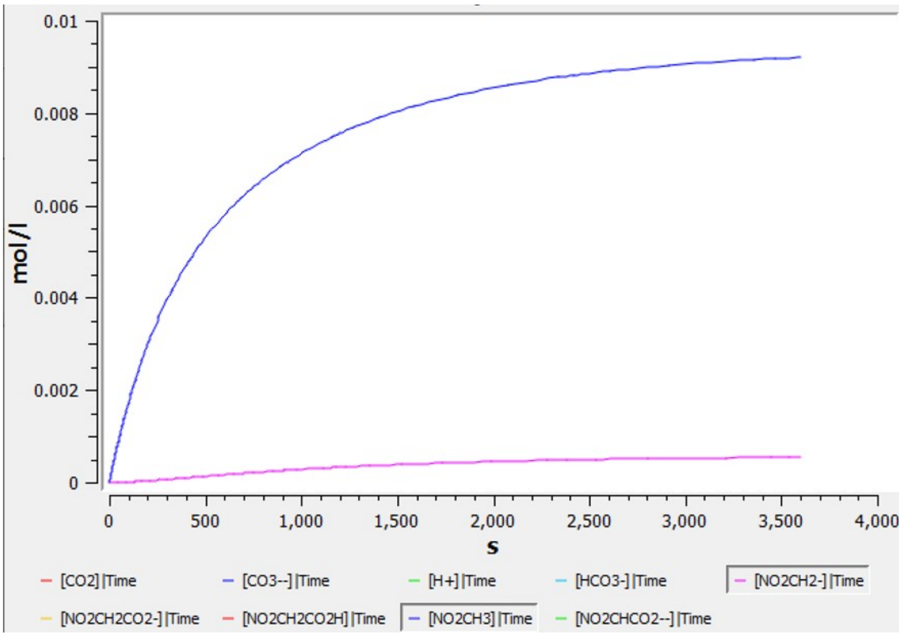

Fig. S2. Plots of the concentrations of nitromethane **5** (blue line) and its conjugate base **4** (violet line) vs. time when 1 equiv. of nitroacetic acid **1** is added to a solution of 0.01 M NaOH at time $t = 0$.

The kinetics of carbon dioxide, hydrogencarbonate ion, and carbonate ion for the title experiment are reported in Fig. S3.

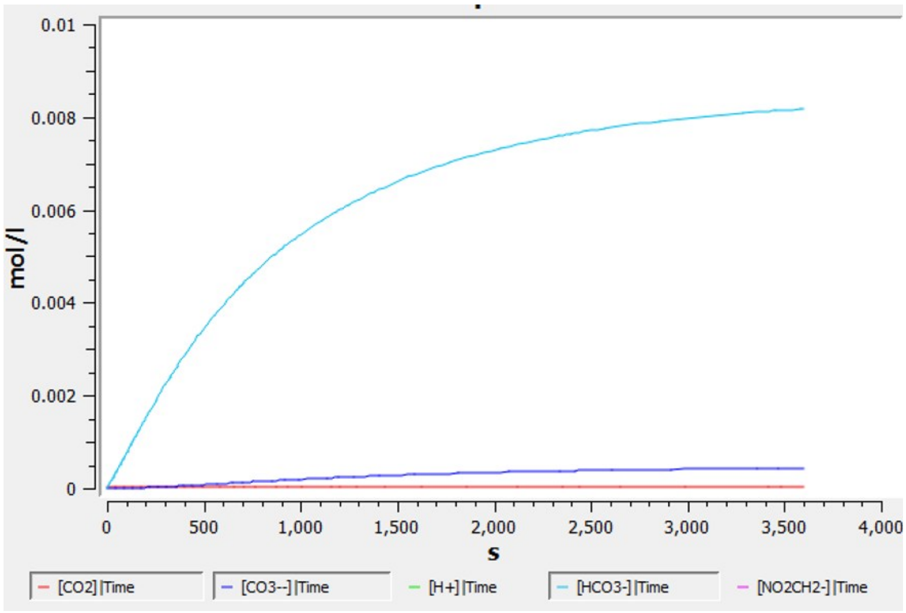

Fig. S3. Plots of the concentrations of carbon dioxide (red line), hydrogencarbonate ion (light blue line), and carbonate ion (blue line) vs. time when 1 equiv. of nitroacetic acid **1** is added to a solution of 0.01 M NaOH at time $t = 0$. Note that the concentration of carbon dioxide is fixed at the constant value of its solubility in water, i.e. $1.63 \cdot 10^{-5}$ M.

Simulated kinetics of 0.010 M NaOH solution added with 2 equivs. of nitroacetic acid 1

Kinetics were simulated with the program COPASI[18] as described in previous section.

In Fig. 1b is reported the plot of pH vs. time when 2 equivs. of nitroacetic acid **1** are added to a solution of 0.01 M NaOH at time $t = 0$. The kinetics of nitroacetic acid **1**, the monoanion **2**, and the dianion **3** for the title experiment are reported in Fig. S4.

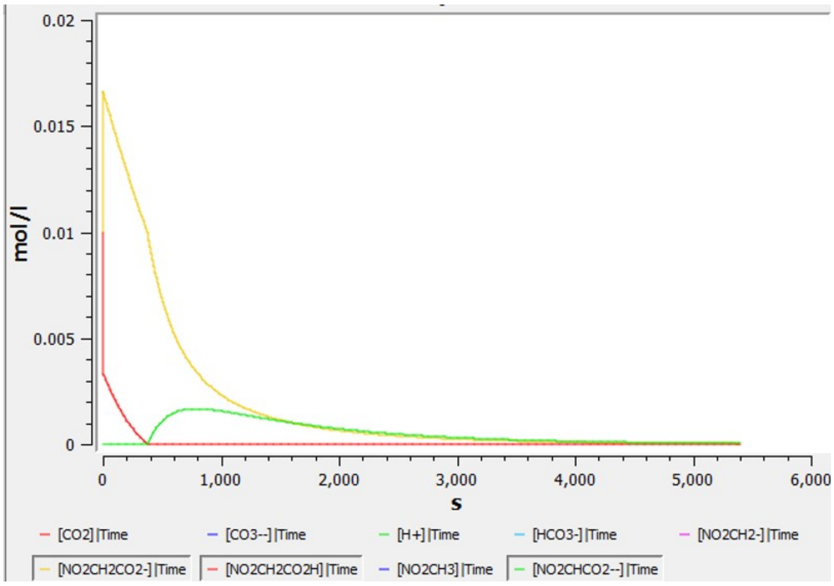

Fig. S4. Plots of the concentrations of nitrocetic acid **1** (red line), the monoanion **2** (yellow line), and the dianion **3** (green line) vs. time when 2 equivs. of nitroacetic acid **1** are added to a solution of 0.01 M NaOH at time $t = 0$.

The kinetics of nitromethane **5**, and its conjugate base **4** for the title experiment are reported in Fig. S5.

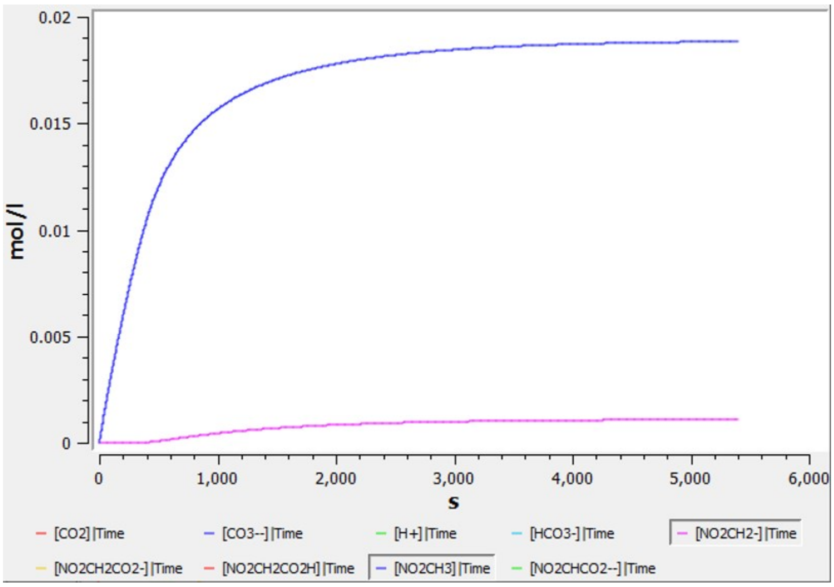

Fig. S5. Plots of the concentrations of nitromethane **5** (blue line) and its conjugate base **4** (violet line) vs. time when 2 equivs. of nitroacetic acid **1** are added to a solution of 0.01 M NaOH at time $t = 0$.

The kinetics of carbon dioxide, hydrogencarbonate ion, and carbonate ion for the title experiment are reported in Fig. S6.

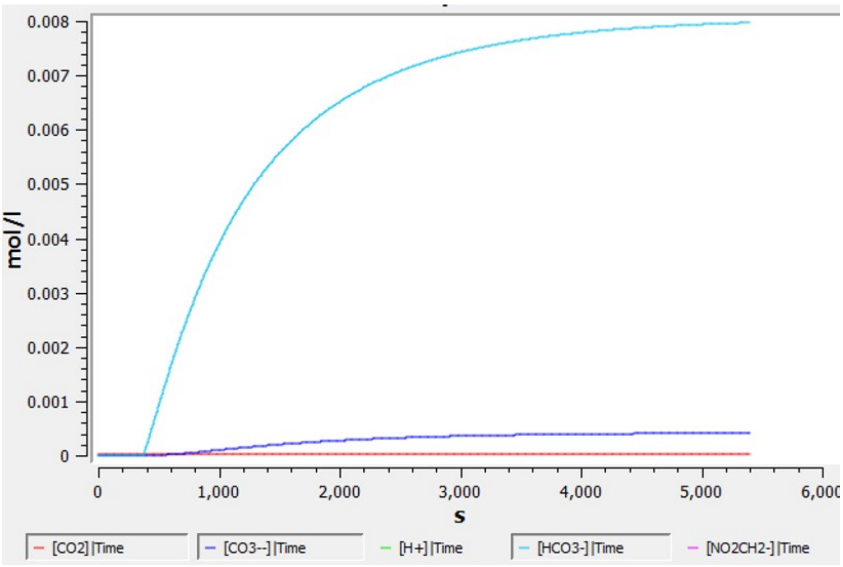

Fig. S6. Plots of the concentrations of carbon dioxide (red line), hydrogencarbonate ion (light blue line), and carbonate ion (blue line) vs. time when 2 equivs. of nitroacetic acid **1** are added to a solution of solution of 0.01 M NaOH at time $t = 0$. Note that the concentration of carbon dioxide is fixed at the constant value of its solubility in water, i.e. $1.63{\cdot}10^{-5}$ M.

Kinetic runs obtained adding 0.015 M acid 1 to 0.0075, 0.015, 0.030 and 0.050 M NaOH

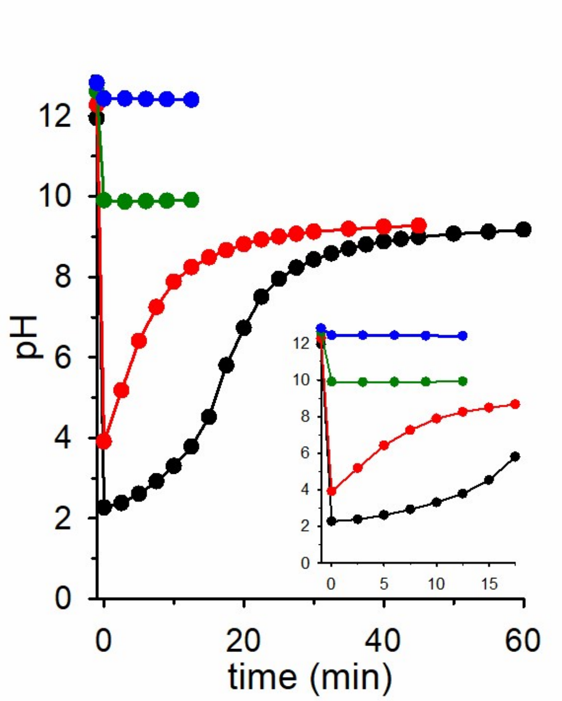

Fig. S7. Plot of pH vs time for the reaction between NaOH at varying concentrations (0.0075 M black, 0.015 M red, 0.030 M green and 0.050 M blue) and **1** (0.015 M). When NaOH is in defect (black) or equimolar (red) to acid **1**, a sigmoidal and monotonical increase to a plateau value is obtained as expected. When NaOH is in excess (green and blue), addition of acid **1** rapidly lowers a little the pH solution. In the latter cases, lowering of pH is due both to the rapid formation of monoanion **2** which quickly decarboxylates to **4** and to the rapid formation of dianion **3**.

pH Monitoring of the Reaction between 0.015 M NaOH and 0.030 M 1 with argon or air bubbling

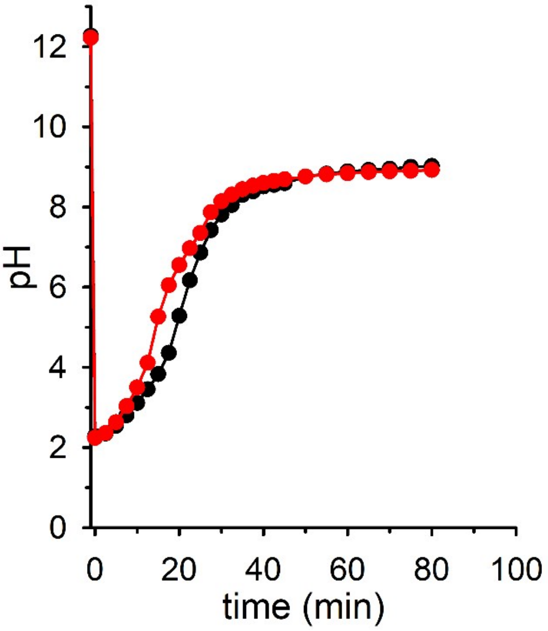

Fig. S8. Plot of pH vs time for the reaction between **1** (0.030 M) and NaOH (0.015 M) with argon (black) and air (red) bubbling. Initial and final pH are the same. The fact that the profiles are not perfectly superimposable is likely due to the difference in the bubbling flux in the two cases (air was bubbled by means of a compressor), which can affect the response time of the microelectrode.

pH Monitoring of the Reaction between 0.010 M NaOH and 0.020M 1 without argon bubbling

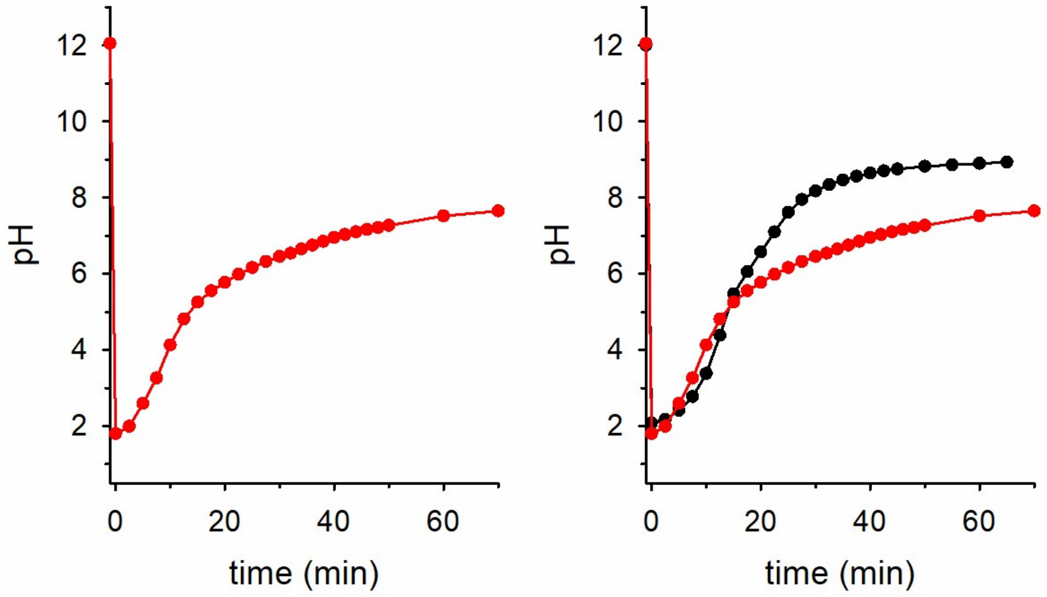

Fig. S9. (left) Plot of pH vs time for the reaction between **1** (0.020 M) and NaOH (0.010 M) without argon bubbling. (right) Comparison between the trace in Fig. S9 left (red) and the pH vs time profile of the reaction carried out under the same conditions with argon bubbling (black). The difference of final pH is approximately 1.

Decarboxylation followed with bromocresol green

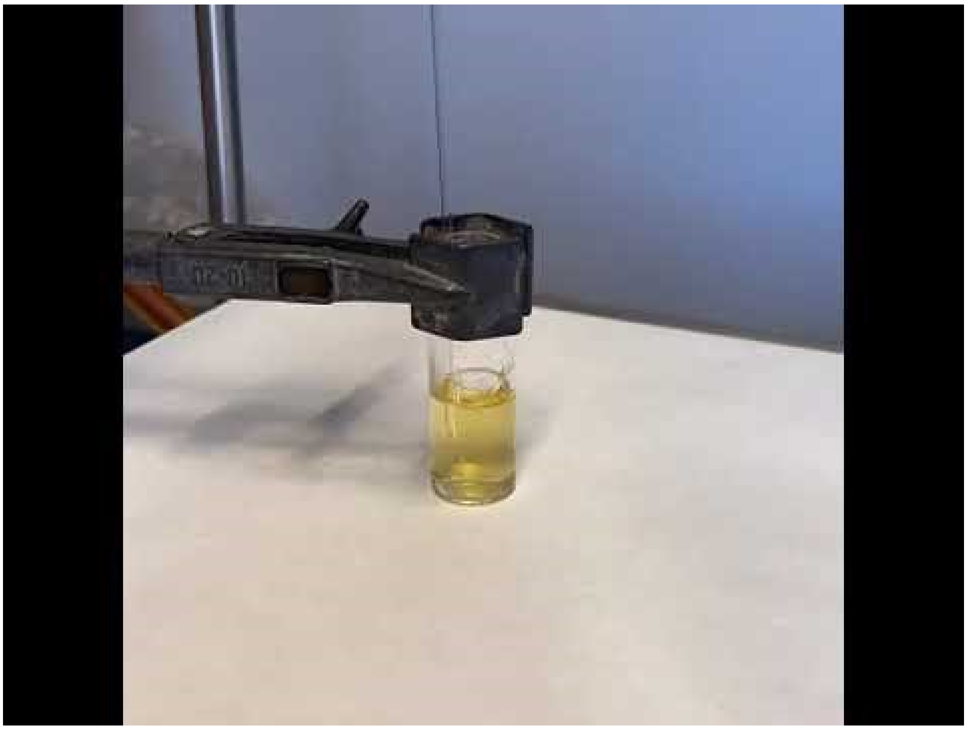

Video S1. Decarboxylation of 0.020 M nitroacetic acid **1** in the presence of 0.010 NaOH (μ= 0.50 M, T= 20°C). Reaction followed by means of bromocresol green as an indicator.

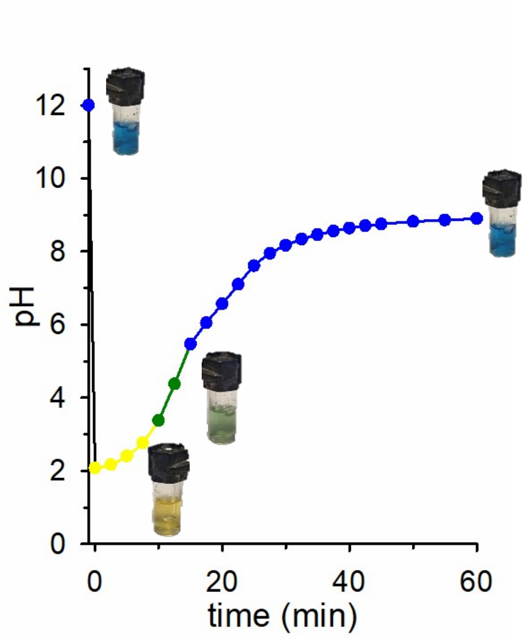

Indicator colors in function of pH:

- Yellow: from pH 1.0 to 3.8
- Green: from pH 3.8 to 5.4
- Blue: from pH 5.4 to 14

Movie speed:

from 1 sec to 11 sec: x1

from 11 sec to 33 sec: x60

A better view of the video is available at the following

Youtube link:

https://youtu.be/XYAt6jevDbg

Decarboxylation followed with methyl red

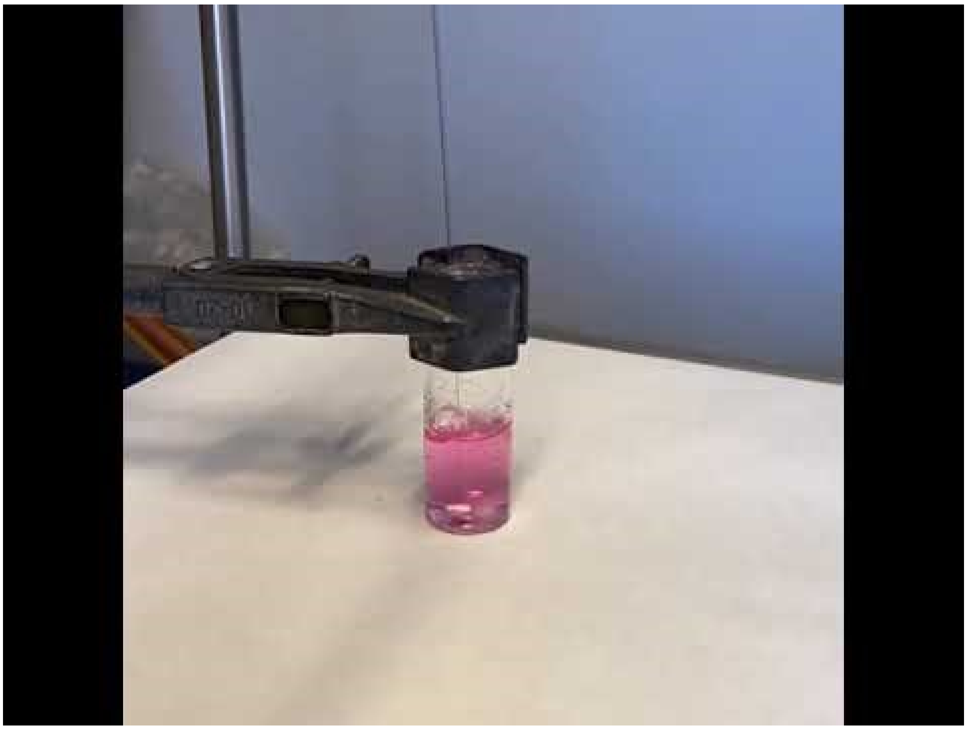

Video S2. Decarboxylation of 0.020 M nitroacetic acid **1** in the presence of 0.010 NaOH (μ= 0.50 M, T= 20°C). Reaction followed by means of methyl red as an indicator.

Indicator colors in function of pH:

- Red: from pH 1.0 to 4.4
- Orange: from pH 4.4 to 6.2
- Yellow: from pH 6.2 to 14

Movie speed:

from 1 sec to 10 sec: x1

from 11 sec to 39 sec: x35

A better view of the video is available at the following Youtube link:

https://youtu.be/CFNbmOtHLhw

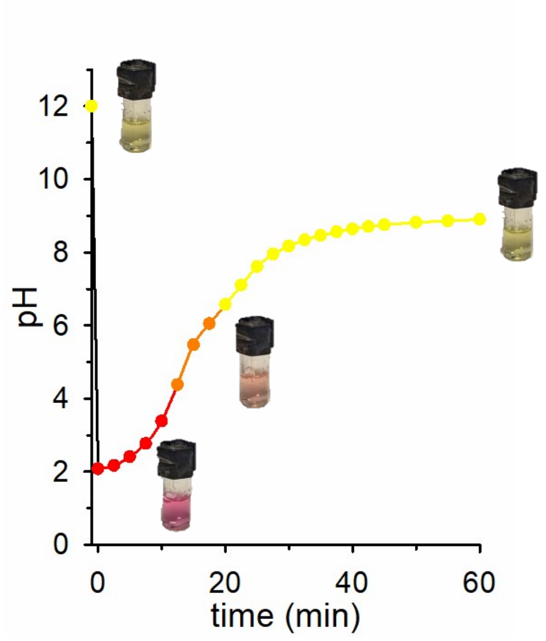

Three $pH_{1(high)}$-$pH_{2(low)}$-$pH_{3(high)}$ sequence triggered by three subsequent fuel pulses without restoring the initial pH (12) after the first two pulses.

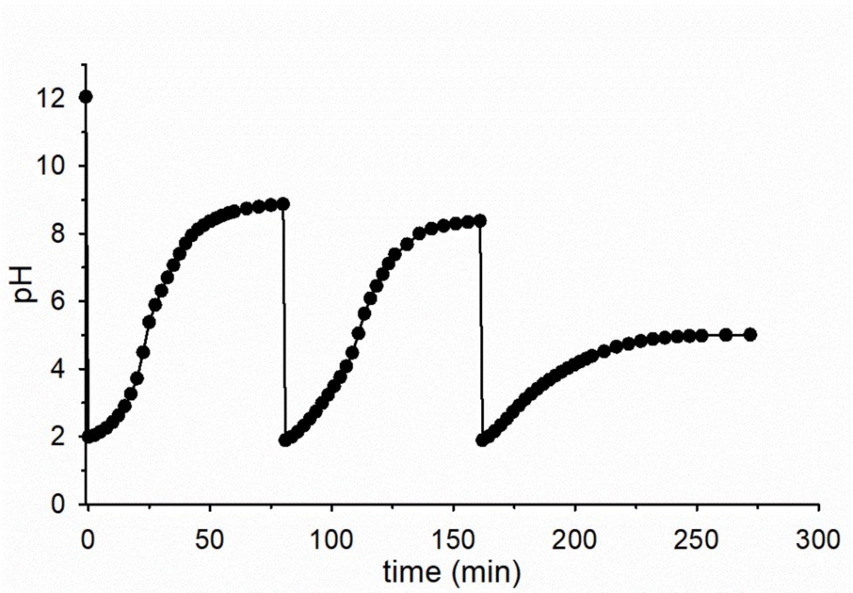

Fig. S10. pH cycles achieved by successive additions of nitroacetic acid. To the initial solution (0.010 M NaOH), aliquots of acid **1** are subsequently added to set the pH at 2 and trigger three pH *high-low-high* cycles (H_2O, T= 20 °C, μ = 0.50 M). Curves are guides for the eye.

A control experiment

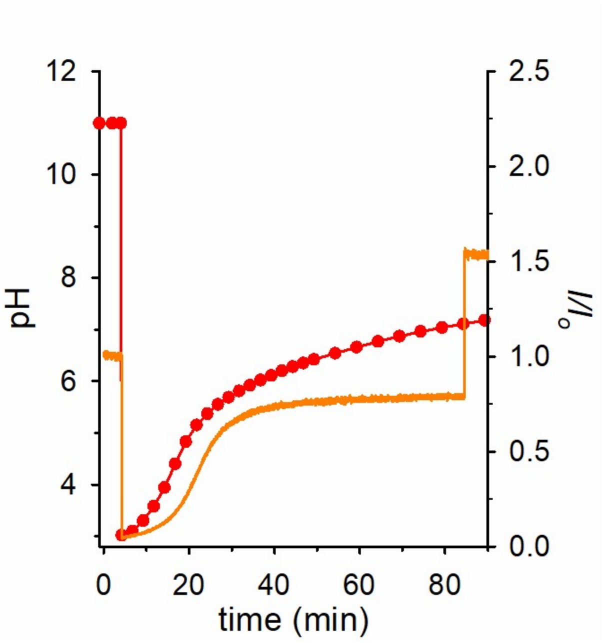

Fig. S11. Time dependence of pH (red trace) and fluorescence emission (orange trace) (λ_{exc} = 282 nm, λ_{em} = 338 nm) of 0.0050 mM **7** (1.0 mM NaOH, μ = 0.50 M; T=20 °C). At t = 4 min, **1** was added (2.0 mM). After monitoring the fluorescence emission for further 80 minutes, **6** was added (2 mM).

Comparison between the fluorescence emission vs time profiles of 0.0050 mM 7 during the decarboxylation of 2.0 mM 1 triggered by 1.0 mM NaOH in the presence or absence of 6

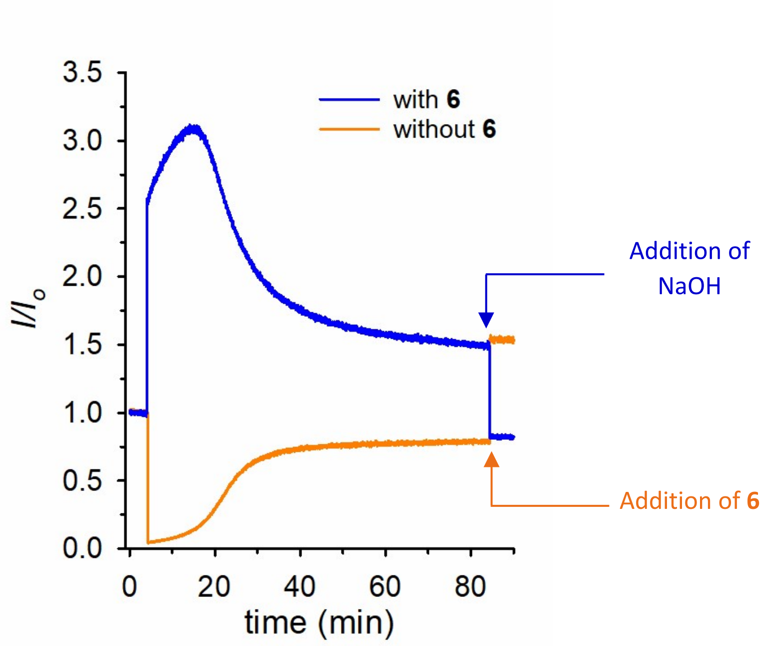

Fig. S12. Comparison between fluorescence emission vs time profiles (λ_{exc} = 282 nm, λ_{em} = 338 nm) recorded during the decarboxylation of 2.0 mM **1** in the presence of 1.0 mM NaOH and 0.0050 mM **7** with (blue trace) or without (orange trace) 2.0 mM **6**. In both samples the initial pH was set to 11.0 (1.0 mM NaOH). At *t* =4 min, to the initial solutions (2.90 mL), 0.100 mL of the stock solution of **1** were added. Then, the variations of fluorescence emission were monitored for further 80 minutes. In one case (blu trace), at *t* =84 min the pH was reset to 11.0 by adding NaOH 1.0 M, in the other case (orange trace), at *t* =84 min the required small volume of a concentrated stock solution of **6** was added.

---

[1] S. F. Vanier, G. Larouche, R. P. Wurz, and A. B. Charette, *Org. Lett.* **2010**, 12, 672-675.
